# Supplementary material for: A model for maxilloturbinate morphogenesis in seals
Source: PLoS One. 2025 Mar 3;20(3):e0316669. doi: 10.1371/journal.pone.0316669 (PMC11875363; doi:10.1371/journal.pone.0316669)
Supplement: Attachment — Submitted filename: rebuttal-letter-2.pdf [file pone.0316669.s002.pdf]

Dear editor and reviewers,

Thank you for the thorough review and comments. The manuscript has been adjusted to reflect Reviewer #1's remaining comments, which are reproduced below in grey alongside detailed replies to the individual points raised. We hope these changes help clarify these shortcomings.

Reviewer #1: There is still a lack of discussion of limitations of the 2D model and its links (whether absent or not) to anisotropic growth mechanisms in 3D. I think it is fine to make assumptions and propose a working principle while the actual biology is not fully understood, as the authors have done here. But this should then at least be discussed appropriately, especially since it is difficult to reconcile the 2D growth model with self-avoidance with a mechanistic way to grow bone tissue anisotropically in 3D. How do the authors picture the growth process in 3D? In 2D it is easy to place new particles at the tips of a tree, but how would the bone sheets grow analogously in 3D? Could the proposed mechanosensing of wall shear stress from fluid flow act as a cue in which direction to grow? I don't expect the authors to have answers to these questions, but they need to be discussed at least, especially since similar ideas on growth anisotropy from wall shear stress sensing in the developing airway have appeared already in the literature (Conrad et al., Development 148, dev194209, 2021).

We agree and have added a more detailed discussion of this aspect (lines 389-403). Specifically, we speculate that elongation along the rostro-caudal axis may be governed by shear stress in a similar fashion to branching in the transverse plane. Thank you for suggesting the Conrad *et al*. paper, which is indeed very relevant. The matter of modelling growth in the rostro-caudal dimension is left as an open question.

Reviewer #1: Moreover, now that the mathematical model description has been revised, it seems that the code implementation does not match the description in the manuscript. From Surface.h, it looks like Newtonian dynamics (a second-order differential equation) is solved with the semi-implicit Euler method. When params.damping = 0, then (essentially)

position += acceleration * dt * dt;

where acceleration is f in the manuscript. From the manuscript, however, one gets a different impression, namely that a discrete form of a first-order differential equation is implemented. Also, I am unable to spot the exponentially decaying term k(t) in the code. There isn't a single call of the exponential function. My guess is that the authors just overdamped their model with supercritical damping coefficients (i.e., small dt in their implementation?), but that would NOT be the same as an exponentially decaying k(t) prefactor in the acceleration or force. k(t)~exp(t/tau) would model plasticity, whereas overdamping models viscosity – two qualitatively different concepts. The description in the manuscript needs to be revised substantially to match what has really been implemented, with a specification of the proper equations of motion and all model parameters used.

The C++ implementation and eq. 1 are in fact equivalent, albeit with potentially confusing variable names in the code. With `params.damping = 0` and `params.dt = 0.5`, at each relaxation timestep the position of each node is updated to (lines 300-303 in Surface.h):

```
particles[i].position + 0.25 * particles[i].acceleration * particles[i].flexibility
```

This corresponds to eq. 1, with $\mathbf{r}_i(t-1)$ = `particles[i].position`, $k(t_i)$ = `0.25 * particles[i].flexibility`, and $\mathbf{f}_i(t-1)$ = `particles[i].acceleration`.

As pointed out however, there is a slight difference between $k(t_i)/K$ and `particles[i].flexibility`. With $K = 0.25$ and $\tau = 4000$, and `params.rigidity` = $1/\tau$, the values of $k(t_i)$ and the implemented "flexibility" ($0.25\ (1-1/\tau)^t$) are almost equal in the relevant range ($t \in [0; 20{,}000]$), with an error of 6.00×10^{-6}. This error has now been corrected and the new version of the simulation code, which matches $k(t_i)$ exactly, has been uploaded to the Figshare repository linked in the manuscript. All simulations have been rerun with this correction applied, and figures and results discussed in the paper now correspond to this corrected version. These new results are very close to those in previous versions of the manuscript, due to the small scale of the error involved (see Table 1 and lines 217-218, 265, 275-278, 287-288). We hope that this was clarifying.

Reviewer #1: This also links to my previous comment that the first manuscript version named the f terms "forces", which they aren't in the formulas presented. This has now been reworded, but only in a minimal sense that will make it even harder for the readers to follow, as the now-called "displacements" are still named f, while the revised manuscript still speaks of "balance between the relevant forces" and "spring forces". This is all subpar and unnecessarily confusing.

Displacement of spring forces have been relabeled **s** instead of **f**, and the model description has been clarified (lines 86-107). Wording has been adjusted to refer to these as displacements or interactions while still conveying that these interactions are governed by spring-like behaviour (lines 70, 73, 93-100). We hope that these changes have made this matter clearer.

Reviewer #1: Coming back to the tree area A_b. I had to look up the code to understand the author's response to my and referee 2's comment on the lack of clarity on how the tree length is converted to an area. I think it is still not clear in the manuscript. Apparently, the actual tree length is used as the occupied area. While this may work numerically, it makes no sense physically, as the units don't match. There is a dimensional proportionality factor missing – even if it is one, in simulation units of length. This should still be specified in the manuscript (potentially in units of r_0, which sets the simulation length scale) to allow the readership to follow what was done.

This is absolutely right, and as pointed out in the first review, a branch thickness does need to be assumed at this point, which matches that used in the calculation of D_h and C_x. This has now been clarified lines 114-119, and the paragraph originally introducing this constant thickness parameter (lines 171-178) has been moved to the end of the description of the relaxation step, lines 127-132.

Reviewer #1: Finally, I still perceive the claims about similarity of distributions in lines 272, 290 as overly strong. I understand that the claims were made based on the observed standard deviations and RMSE. But this is insufficient to claim similarity. Two distributions can be vastly different yet have the same standard deviations. Why not just comment on similarity of the standard deviations and/or means, rather than the entire distributions? Such a claim would then be justified.

These claims have been reworded in accordance with the suggestion (lines 257-259 and 277).

Thank you once again for these comments and for your consideration.

Best regards,

Jonathan Kings
